# Effects of Nitrogen Application Rate on Nitrogen Uptake and Utilization in Waxy Sorghum Under Waxy Sorghum–Soybean Intercropping Systems

**DOI:** 10.3390/plants14091384

**Published:** 2025-05-03

**Authors:** Can Wang, Siyu Chen, Fangli Peng, Qiang Zhao, Jie Gao, Lingbo Zhou, Guobing Zhang, Mingbo Shao

**Affiliations:** Institute of Upland Food Crops, Guizhou Academy of Agricultural Sciences, Guiyang 550006, China; wangc.1989@163.com (C.W.); hlscsy1995@163.com (S.C.); Pfl9367@126.com (F.P.); 15761633106@163.com (Q.Z.); gaojie396300520@163.com (J.G.); 85103@163.com (L.Z.); GZzgb1990@126.com (G.Z.)

**Keywords:** intercropping, nitrogen fertilizer, waxy sorghum, nitrogen use efficiency, yield

## Abstract

Waxy sorghum–soybean intercropping is a sustainable and intensive farming system in southwest China. However, there is limited knowledge about the effects of intercropped soybean combined with nitrogen application on nitrogen uptake and utilization in waxy sorghum. A two-year (2023 and 2024) field experiment was carried out using a randomized complete block design with three planting patterns and three nitrogen application rates to explore the responses of grain yield formation and nitrogen uptake, accumulation, transportation, metabolism physiology, and utilization of waxy sorghum for intercropped soybean combined with nitrogen application. Planting patterns included sole cropped waxy sorghum (SCW), sole cropped soybean (SCS), and waxy sorghum intercropped with soybean (WSI), and nitrogen application rates included zero nitrogen (N0), medium nitrogen (N1), and high nitrogen (N2). Results showed that the dry matter accumulation amount, nitrogen content, nitrogen accumulation amount, nitrogen transportation amount, nitrogen transportation rate, contribution rate of nitrogen transportation to grains, nitrogen metabolizing enzymes activities (including nitrate reductase, nitrite reductase, glutamine synthetase, glutamate synthetase, glutamate dehydrogenase, and glutamic-pyruvic transaminase), and active substances contents (including soluble sugar, soluble protein, and free amino acid) in various organs of waxy sorghum among planting patterns and nitrogen application rates were in the order of WSI > SCW and N1 > N2 > N0, respectively. In addition, the nitrogen uptake efficiency, nitrogen agronomy efficiency, nitrogen apparent efficiency, nitrogen recovery efficiency, nitrogen partial factor productivity, and nitrogen contribution rate of waxy sorghum among planting patterns and nitrogen application rates were in the sequence of WSI > SCW and N1 > N2, respectively. The changes in above traits resulted in the WSI-N1 treatment obtaining the highest grain yield (6020.66 kg ha^−1^ in 2023 and 6159.81 kg ha^−1^ in 2024), grain weight per spike (65.22 g in 2023 and 64.51 g in 2024), 1000-grain weight (23.14 g in 2023 and 23.18 g in 2024) of waxy sorghum, and land equivalent ratio (1.41 in 2023 and 1.44 in 2024). Overall, waxy sorghum intercropped with soybean combined with medium nitrogen application (220 kg ha^−1^ for waxy sorghum and 18 kg ha^−1^ for soybean) can help enhance the nitrogen uptake and utilization of waxy sorghum by improving nitrogen metabolizing enzymes’ activities and active substances’ contents, thereby promoting its productivity.

## 1. Introduction

Nitrogen is one of the most important mineral elements to promote plant growth and increase crop yield, and it is also a key limiting factor affecting physiological metabolism and growth of crops in organic and conventional agricultural production [1,2]. As a life element of plant growth, nitrogen is involved in a series of physiological metabolic processes in plants, such as nucleic acid and protein metabolism, synthesis of enzymes and chlorophyll, cell division and differentiation, and hormone regulation [3,4]. Nitrogen metabolism is one of the basic physiological processes of plants, which is mainly catalyzed by a series of enzymes (e.g., nitrate reductase, nitrite reductase, glutamine synthetase, glutamate synthase, glutamate dehydrogenase, and glutamic-pyruvic transaminase) and regulated by various active substances (e.g., soluble sugar, soluble protein, and free amino acid) [5,6,7]. Nitrogen uptake and utilization by crops is a key process of the nitrogen cycle in an agroecological system and an important basis for crop yield formation [8]. Therefore, determining the characteristics of plant nitrogen uptake and utilization is key to improving crops’ nitrogen use efficiency.

The world population is expected to rise to 9.7 billion in 2050, which will lead to serious food security problems [9]. There is an urgent need to increase crop yields to meet the daily food consumption of such a large global population. Nitrogen application is an important agricultural measure to maintain food production and ensure food security, and its contribution rate to the increase in crop yield can reach about 40% [10]. Numerous studies have shown that reasonable nitrogen application can improve roots’ architecture, enhance leaves’ photosynthetic capacity, promote the absorption of nitrogen by plants, accelerate plant growth, and increase nitrogen use efficiency and crop yield [11,12,13]. However, excessive nitrogen application has a negative impact on agricultural production, not only not increasing crop yield, but also reducing nitrogen use efficiency and producing environmental problems, such as an increase in greenhouse gas emissions, acidification of farmland soil, and imbalance of the soil ecosystem [14,15]. Consequently, reducing agricultural non-point source pollution and optimizing nitrogen fertilizer management measures to improve nitrogen use efficiency are key to achieving sustainable agricultural development.

In addition to nitrogen application, planting patterns are also an important factor affecting nitrogen utilization and crop yield [16]. Intercropping refers to a planting pattern in which two or more crops are grown in the same field during the same growing season, which can make better use of light, temperature, water, gas, and nutrients to increase resource use efficiency and land productivity [17,18]. As a recognized sustainable agricultural production system, cereal–legume intercropping systems has been widely implemented in various countries worldwide to ensure global food security [19]. In cereal–legume intercropping systems, legume crops can not only reduce the input of chemical nitrogen through biological nitrogen fixation but also transfer a certain amount of nitrogen to neighboring cereal crops, thus providing additional nitrogen sources for the growth and development of cereal crops [20,21]. In addition, intercropping of cereal and legume crops can improve the ventilation and light transmission conditions of tall-statured crops through the tall and dwarf configuration of crops, thus increasing its photosynthetic capacity [22]. In addition, cereal–legume intercropping systems can promote nitrogen uptake by crops by coordinating interspecific competition, effectively improving the nitrogen use efficiency and yield of crops as well as land productivity [23]. Furthermore, cereal–legume intercropping systems are environmentally friendly by improving the soil environment and reducing nitrogen leaching and greenhouse gas emissions [24,25]. Nitrogen complementary use is the main reason for intercropping’s advantage, and nitrogen application can regulate the interspecific relationship between cereal and legume crops by altering the vegetative growth of cereal crops and the symbiotic nitrogen fixation ability of legume crops in cereal–legume intercropping systems [26,27]. Thus, determining the responses of crops to nitrogen in cereal–legume intercropping systems is crucial to improve its intercropping advantage.

Waxy sorghum belongs to the grain sorghum in sorghum taxonomy according to different purposes, and is widely adopted as a raw material for brewing liquor (e.g., Moutai, Wuliangye, Xijiu, Langjiu, and Luzhoulaojiao) in China due to its high amylopectin content [28]. In particularly, Guizhou Province has the largest planting area of waxy sorghum in China, with about 200,000 hectares per year, which mainly support the development of Moutai-flavor liquor enterprises. However, due to the limited cultivated land in Guizhou Province, the decreases in yield and quality caused by waxy sorghum continuous cropping have seriously restricted the development of Moutai-flavor liquor enterprises. To alleviate the obstacles of waxy sorghum continuous cropping, we have carried out intercropping research and found that waxy sorghum intercropped with soybean improved rhizosphere soil quality, enhanced leaves photosynthetic capacity, and promoted the accumulation of dry matter and nutrients in waxy sorghum [29,30,31]. Nevertheless, there is limited knowledge about the effects of intercropped soybean combined with nitrogen application on the nitrogen uptake characteristics in waxy sorghum, and the nitrogen utilization mechanism in waxy sorghum–soybean intercropping systems remains unclear. We hypothesized that intercropped soybean combined with reasonable nitrogen application can increase nitrogen use efficiency by promoting nitrogen uptake, accumulation, and metabolism of waxy sorghum. The objective of this study was to (1) quantify the effects of intercropped soybean combined with nitrogen application on the nitrogen uptake, accumulation, and metabolism in waxy sorghum, and (2) determine the nitrogen utilization mechanism in waxy sorghum–soybean intercropping systems. This study will provide a theoretical basis and technical support for nitrogen fertilizer management in waxy sorghum–soybean intercropping systems.

## 2. Materials and Methods

### 2.1. Experimental Site and Materials

A two-year (2023 and 2024) field experiment was carried out in Guiyang Experimental Station (26°32′ N, 106°48′ E) of Guizhou Academy of Agricultural Sciences (Guiyang, China) with an altitude of 1139 m. The experimental site has a subtropical humid monsoon climate and the daily mean air temperature and precipitation during the two growing seasons are shown in Figure S1. The soil type is sandy loamy with a pH of 7.68, organic matter of 33.47 g kg^−1^, total N of 1.68 g kg^−1^, total P of 0.94 g kg^−1^, total K of 9.75 g kg^−1^, available N of 104.33 mg kg^−1^, available P of 18.61 mg kg^−1^, and available K of 209.16 mg kg^−1^ in the 0–200 mm soil layer at the start of the experiment in 2023, which is classified as a medium to high fertility level according to the classification standards of cultivated land quality grades in China.

The waxy sorghum cultivar Hongliangfeng-1 and soybean strain Yindou-1 were used in the experiment. Hongliangfeng-1 is a brewing-type sorghum cultivar with a semi-compact plant type, and its appropriate fertilization strategy is 200 kg ha^−1^ N, 100 kg ha^−1^ P_2_O_5_, and 300 kg ha^−1^ K_2_O. Yindou-1 is a grain-type spring soybean strain with a compact plant type, determinate podding habit, yield potential of 2400 kg ha^−1^, and average protein content of 41.25%, and its appropriate fertilization strategy is 60 kg ha^−1^ N, 60 kg ha^−1^ P_2_O_5_, and 40 kg ha^−1^ K_2_O. The seeds of waxy sorghum and soybean were provided by the Institute of Upland Food Crops, Guizhou Academy of Agricultural Sciences (Guiyang, China). Nitrogen fertilizer (urea containing 46.2% N) was obtained from Gzuizhou Chitianhua Tongzi Chemical Co., Ltd. (Zunyi, China). Phosphate fertilizer (calcium superphosphate containing 12% P_2_O_5_) was obtained from Guizhou Qiantianhua Ecological Fertilizer Co., Ltd. (Fuquan, China). Potassium fertilizer (potassium sulfate containing 52% K_2_O) was obtained from SDIC Xinjiang Luobupo Potash Co., Ltd. (Ruoqiang, China).

### 2.2. Experimental Design and Crop Management

The field experiment was conducted using a randomized complete block design with three planting patterns and three nitrogen application rates. Planting patterns included sole cropped waxy sorghum (SCW), sole cropped soybean (SCS), and waxy sorghum intercropped with soybean (WSI), and nitrogen application rates included zero nitrogen (N0), medium nitrogen (N1), and high nitrogen (N2). The detailed fertilization strategies for experimental treatments are presented in Table 1. For the SCW treatment, the plot size was 17.5 m^2^ (5 m long and 3.5 m wide) with row spacing of 70 cm and hill spacing of 25 cm, and consisted of five waxy sorghum planting rows. For the SCS treatment, the plot size was 12.5 m^2^ (5 m long and 2.5 m wide) with row spacing of 50 cm and hill spacing of 25 cm, and consisted of five soybean planting rows. For the WSI treatment, the plot size was 25.5 m^2^ (5 m long and 5.1 m wide) with a row ratio configuration of two rows of waxy sorghum intercropped with one row of soybean with a bandwidth of 140 cm, waxy sorghum row spacing of 40 cm, distance between adjacent waxy sorghum and soybean rows of 50 cm, waxy sorghum hill spacing of 25 cm, and soybean hill spacing of 25 cm, consisting of eight waxy sorghum planting rows and three soybean planting rows. All treatments included six replicates, of which three replicates were used for sampling, and another three replicates were used for yield measurement.

The soil was left idle in winter before the experiment began and plowed with a rotary tiller to a depth of 25–30 cm about 15 days before sowing. Waxy sorghum was artificially sown on 14 April 2023 and 12 April 2024, thinned at the four-leaf stage to a uniform specification of two plants per hill, and artificially harvested on 21 August 2023 and 22 August 2024, respectively. Soybean was artificially sown on 14 April 2023 and 12 April 2024, thinned at the four-leaf stage to a uniform specification of two plants per hill, and artificially harvested on 28 July 2023 and 27 July 2024, respectively. Nitrogen, phosphate, and potassium fertilizers were mixed and then applied into the soil as basal fertilizer when the seeds were sown. Phoxim (granules with effective constituent of 3%, Leshan Xinlu Chemical Co., Ltd., Leshan, China) was mixed with basal fertilizer at a dose of 12 kg ha^−1^ to control underground pests (e.g., grub, mole cricket, and cutworm). Imidacloprid (wettable powder with an effective constituent of 10%, Jiangsu Kangpeng Agrochemical Co., Ltd., Taizhou, China) and carbendazim (wettable powder with an effective constituent of 50%, Sichuan Guoguang Agrochemical Co., Ltd., Chengdu, China) were, respectively, diluted 500 times with water and applied via foliar spraying at the seedling stage of waxy sorghum and soybean to prevent insects (e.g., aphid and borer of waxy sorghum, and aphid, busck, and clanis bilineata walker of soybean) and diseases (e.g., rust disease, red leaf disease, anthracnose of waxy sorghum, root rot, mosaic virus disease, and rust disease of soybean). Artificial weeding was performed at the seedling stage of waxy sorghum and the branching stage of soybean.

### 2.3. Measurements and Calculations

#### 2.3.1. Measurements of Dry Matter Accumulation

According to the method described by Wang et al. [29], with slight modification, at the anthesis stage and maturity stage, three waxy sorghum plants were selected randomly from the middle strip of each plot. The selected plants were uprooted with a small hoe according to the specification with a radius of 10 cm centered on the plant and a soil depth of 60 cm, and the roots were washed clean with a slow water flow. Then, the roots, culms, leaves, spikes, and grains were separated technically, according to the growth characteristics of each organ in waxy sorghum. Every organ was placed in an oven (Jiangdong DHG-9240A, Suzhou Jiangdong Precision Instrument Co., Ltd., Suzhou, China) for 30 min at 105 °C to kill the fresh tissues and then dried to constant weight at 80 °C. The dry weight of each organ was measured with an electronic balance (XingYun JA203H, Changzhou Xingyun Electronic Equipment Co., Ltd., Changzhou, China) and the dry matter accumulation amount (DMA) was converted according to the number of waxy sorghum plants and the area in each plot.

#### 2.3.2. Determinations of Nitrogen Accumulation and Transportation

After the measurement of dry weight, the dried sample of each organ was sent to Guizhou Bailuoni Testing Technology Co., Ltd. (Guiyang, China) to determine the nitrogen content (NC) using the Kjeldahl method. The nitrogen accumulation amount (NA), nitrogen transportation amount before anthesis (NTA), nitrogen transportation rate before anthesis (NTR), and contribution rate of nitrogen transportation before anthesis to grains (GCRNT) in waxy sorghum were calculated as the following formulas described by Wang et al. [30]:

NA = DMA × NC

NTA = NAA − NAM

NTR = NTA/NAA × 100%

GCRNT = NTA/GNA × 100%

where NAA is the nitrogen accumulation amount of the vegetative organ at the anthesis stage, NAM is the nitrogen accumulation amount of the vegetative organ at the maturity stage, and GNA is the nitrogen accumulation amount of grains at the maturity stage.

#### 2.3.3. Measurements of Nitrogen Metabolism Physiology

Three waxy sorghum plants were selected randomly from the middle strip of each plot at the anthesis stage and maturity stage, and the third functional leaves from the top of each plant was used to measure the enzyme activities and contents of active substances related to nitrogen metabolism using the kit produced by Beijing Solarbio Science and Technology Co., Ltd. (Beijing, China) following the instructions of the manufacturer.

In brief, the nitrate reductase (NR) activity was assayed using the Griess colorimetric method and one unit of enzyme activity (U) was defined as 1 μmol of NO_2_^−^ generated by 1 g of leaves sample per hour. The nitrite reductase (NiR) activity was determined using visible spectrophotometry and one unit of enzyme activity (U) was defined as 1 μmol of NO_2_^−^ reduced by 1 g of leaves sample per hour. The glutamine synthetase (GS) activity was measured using the visible spectrophotometry and one unit of enzyme activity (U) was defined as a change in absorbance of 0.01 at 540 nm caused by 1 g of leaves sample per minute in the 1 mL reaction system. The glutamate synthetase (GOGAT) and glutamate dehydrogenase (GDH) activities were assayed using the ultraviolet spectrophotometry and one unit of enzyme activity (U) was defined as 1 nmol NADH consumed by 1 g of leaves sample per minute. The glutamic-pyruvic transaminase (GPT) was determined using the visible spectrophotometry and one unit of enzyme activity (U) was defined as 1 μmol pyruvic acid generated by 1 g of leaves sample per hour. In addition, the soluble protein (SP) content was measured using the Coomassie brilliant blue method, and soluble sugar (SS) and free amino acid (FAA) contents were determined using visible spectrophotometry.

#### 2.3.4. Determinations of Yield, Yield Components, and Land Equivalent Ratio (LER)

At the maturity stage, five waxy sorghum plants were selected randomly from the middle strip of each plot to measure the grain number per spike and 1000-grain weight. Next, all waxy sorghum and soybean plants in each plot were hand-harvested and the grain yields of waxy sorghum and soybean were determined, respectively. The LER was calculated as the following formula described by Wang et al. [29]:

LER = GY_iw_/GY_sw_ + GY_is_/GY_ss_

where GY_iw_ is the grain yield of intercropped waxy sorghum, GY_sw_ is the grain yield of sole cropped waxy sorghum, GY_is_ is the grain yield of intercropped soybean, and GY_ss_ is the grain yield of sole cropped soybean.

#### 2.3.5. Calculations of Nitrogen Use Efficiency

The nitrogen uptake efficiency (NUE), nitrogen agronomy efficiency (NAE), nitrogen apparent efficiency (NAPE), nitrogen recovery efficiency (NRE), nitrogen partial factor productivity (NPFP), and nitrogen contribution rate (NCR) in waxy sorghum were calculated as the following formulas described by Antille and Moody [32], Coêlho et al. [33], and Zheng et al. [34]:

NUE = TNAP/NAR

NAE = (GYNP − GYNNP)/NAR

NAPE = (NAGNP − NAGNNP)/NAR × 100%

NRE = (TNAPNP − TNAPNNP)/NAR × 100%

NPFP = GYNP/NAR

NCR = (GYNP − GYNNP)/GYNP

where TNAP is the total nitrogen accumulation amount of plants, NAR is the nitrogen application rate, GYNP is the grain yield in nitrogen application, GYNNP is the grain yield in no nitrogen application, NAGNP is the nitrogen accumulation amount of grains in nitrogen application, NAGNNP is the nitrogen accumulation amount of grains in no nitrogen application, TNAPNP is the total nitrogen accumulation amount of plants in nitrogen application, and TNAPNNP is the total nitrogen accumulation amount of plants in no nitrogen application.

### 2.4. Statistical Analysis

Data were organized and converted using Microsoft Excel 2021 software (Microsoft Corp., Redmond, WA, USA). Two-way analysis of variance (ANOVA) was performed with DPS v7.05 software (Hangzhou Ruifeng Information Technology Co., Ltd., Hangzhou, China) to evaluate the effects of the planting pattern and nitrogen application rate on each index. The planting pattern, nitrogen application rate, and their interaction were treated as fixed factors, and replicates (*n* = 3) were treated as random factors. Then, the least significant difference (LSD) method was used to test the significance of differences among treatments, and the significance level was set as *p* < 0.05. Partial least squares path modeling (PLS-PM) analysis was performed to explore the relationships among planting pattern and nitrogen application rate with nitrogen uptake, nitrogen accumulation, nitrogen transportation, nitrogen metabolism physiology, nitrogen utilization, and yield formation in waxy sorghum by SmartPLS 4.0.1.3 software (SmartPLS GmbH, Bönningstedt, Germany). All statistical analyses were concentrated on a period of time (about 15 days) and conducted with the same person. Finally, figures were drawn by SigmaPlot 12.5 software (Aspire Software Intl., Ashburn, VA, USA) and embellished by Adobe Illustrator 2023 software (Adobe Systems Inc., San Jose, CA, USA).

## 3. Results

### 3.1. DMA

**Figure 1:**
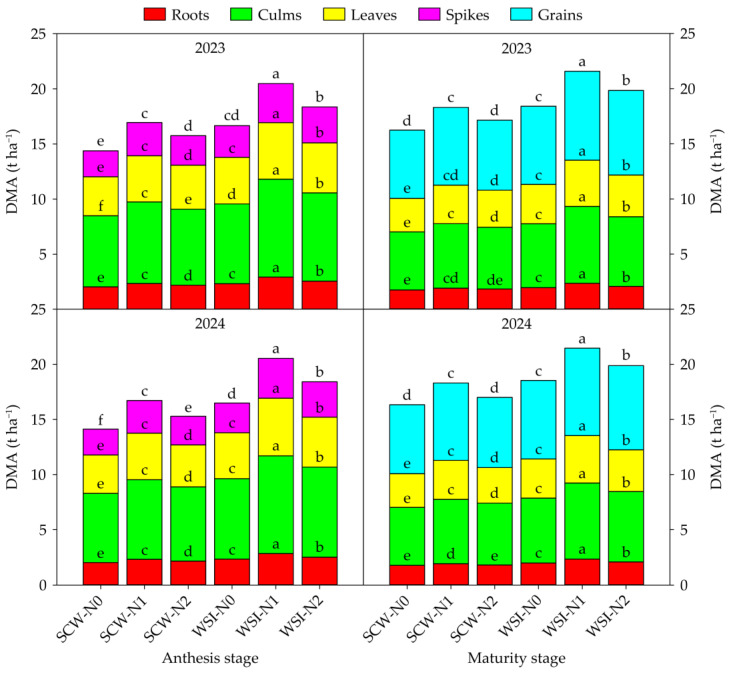
Effects of planting pattern and nitrogen application rate on the dry matter accumulation amount (DMA) in waxy sorghum. Data are the mean of three replicates and different lowercase letters within an organ in the same growth stage and year indicate significant differences among treatments at the 0.05 level. SCW: Sole cropped waxy sorghum; WSI: Waxy sorghum intercropped with soybean; N0: Zero nitrogen; N1: Medium nitrogen; N2: High nitrogen.

The planting pattern and nitrogen application rate had a significant effect on the DMA of each organ in both stages and both years. The interaction between planting pattern and nitrogen application rate had a significant effect on the DMA, except for the spikes at the anthesis stage and leaves and grains at the maturity stage in 2023 (Table S1). Across years and treatments, the order of DMA among organs was culms > leaves > spikes > roots at the anthesis stage and grains > culms > leaves > roots at the maturity stage, respectively (Figure 1). In both stages and both years, the DMA of each organ under the WSI treatment was higher than that under the SCW treatment. In addition, the DMA of each organ increased with the increase in nitrogen application rate from N0 to N1, while the DMA decreased with the increase in nitrogen application rate from N1 to N2.

### 3.2. Nitrogen Accumulation and Transportation

The NC of each organ was significantly affected by planting pattern and nitrogen application rate in both stages and both years. However, the interaction between planting pattern and nitrogen application rate only had significant effects on the NC of roots in both stages in 2023, leaves in both stages in 2024, and spikes at the anthesis stage in both years (Table 2). In both years and all treatments, the NC among organs was in the sequence of leaves > spikes > roots > culms at the anthesis stage and leaves > grains > roots > culms at the maturity stage, respectively. In addition, the NC of each organ among planting patterns and nitrogen application rates was, respectively, in the order of WSI > SCW and N1 > N2 > N0 in both stages and both years.

**Figure 2:**
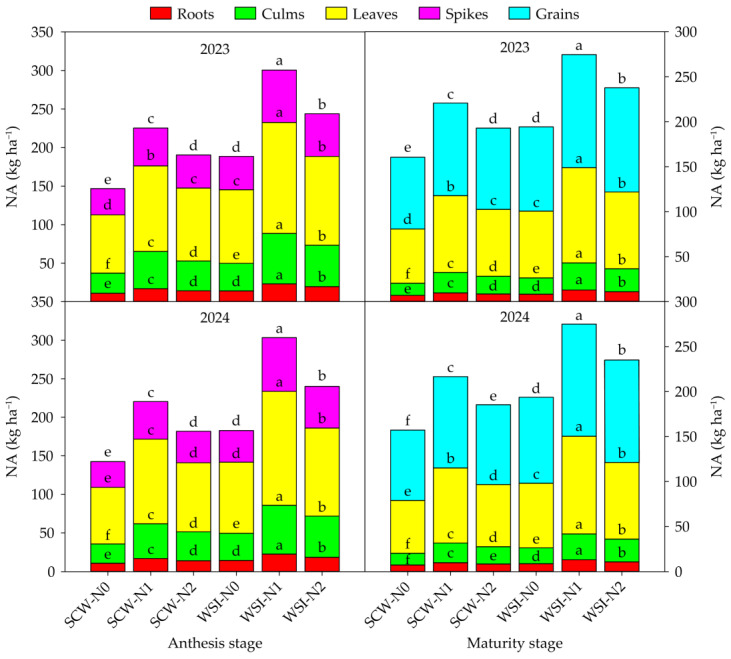
Effects of planting pattern and nitrogen application rate on the nitrogen accumulation amount (NA) in waxy sorghum. Data are the mean of three replicates and different lowercase letters within an organ in the same growth stage and year indicate significant differences among treatments at the 0.05 level. SCW: Sole cropped waxy sorghum; WSI: Waxy sorghum intercropped with soybean; N0: Zero nitrogen; N1: Medium nitrogen; N2: High nitrogen.

The planting pattern, nitrogen application rate, and their interaction had significant effect on the NA of each organ in both stages and both years (Table S2). Across years and treatments, the NA among organs was in the order of leaves > spikes > culms > roots at the anthesis stage and grains > leaves > culms > roots at the maturity stage, respectively (Figure 2). In both stages and both years, the NA of each organ under the WSI treatment was higher than that under the SCW treatment. Moreover, the NA of each organ increased with the increase in the nitrogen application rate from N0 to N1, while decreased with the increase in the nitrogen application rate from N1 to N2.

The NTA, NTR, and GCRNT of each organ were significantly affected by the planting pattern and nitrogen application rate in both stages and both years. However, the interaction between planting pattern and nitrogen application rate only had significant effects on the NTA of roots and culms in 2023, NTA of culms and leaves in 2024, and NTR of roots in 2024 (Table 3). In both years and all treatments, the NTA, NTR, and GCRNT among organs were in the sequence of leaves > culms > roots, culms > roots > leaves, and leaves > culms > roots, respectively. For the mean of two years, compared with the SCW treatment, the WSI treatment increased the NTA, NTR, and GCRNT by, respectively, 43.55%, 7.69%, and 16.34% for roots, 46.01%, 4.95%, and 18.04% for culms, and 44.72%, 12.86%, and 16.69% for leaves. In addition, compared to the N0 treatment, the N1 and N2 treatments increased the mean NTA of two years by, respectively, 82.19% and 45.89% for roots, 96.67% and 55.80% for culms, and 81.50% and 39.64% for leaves, increased the mean NTR of two years by, respectively, 15.10% and 10.80% for roots, 8.39% and 3.53% for culms, and 18.82% and 13.31% for leaves, and increased the mean GCRNT of two years by, respectively, 39.57% and 24.20% for roots, 50.20% and 31.94% for culms, and 38.17% and 18.38% for leaves.

### 3.3. Nitrogen Metabolism Physiology

The activities of NR (Table S3), NiR (Table S4), and GS (Table S5) in each organ were significantly affected by planting pattern and nitrogen application rate in both stages and both years but were less influenced by interaction between planting pattern and nitrogen application rate. Across years and treatments, the orders of NR, NiR, and GS activities among organs were leaves > spikes > roots > culms at the anthesis stage. At the maturity stage, the NR activity among organs was in the order of leaves > roots > grains > culms, and the NiR and GS activities among organs were in the sequence of leaves > grains > roots > culms in both years and all treatments. In addition, the NR, NiR, and GS activities of each organ among planting patterns and nitrogen application rates were, respectively, in the order of WSI > SCW and N1 > N2 > N0 in both stages and both years.

The planting pattern and nitrogen application rate had significant effects on the GOGAT (Figure S2), GDH (Figure S3), and GPT (Figure S4) activities of each organ in both stages and both years, and the interaction between planting pattern and nitrogen application rate also affected the activities of these three enzymes in each organ. In both years and all treatments, the activities of GOGAT, GDH, and GPT among organs were, respectively, in the sequence of spikes > roots > culms > leaves, spikes > leaves > roots > culms, and leaves > spikes > roots > culms at the anthesis stage, and were, respectively, in the order of grains > leaves > roots > culms, leaves > roots > grains > culms, and leaves > culms > roots > grains at the maturity stage. In both stages and both years, the GOGAT, GDH, and GPT activities of each organ under the WSI treatment were higher than those under the SCW treatment. Furthermore, the activities of GOGAT, GDH, and GPT in each organ increased with the increase in the nitrogen application rate from N0 to N1, and decreased with the increase in the nitrogen application rate from N1 to N2.

**Figure 3:**
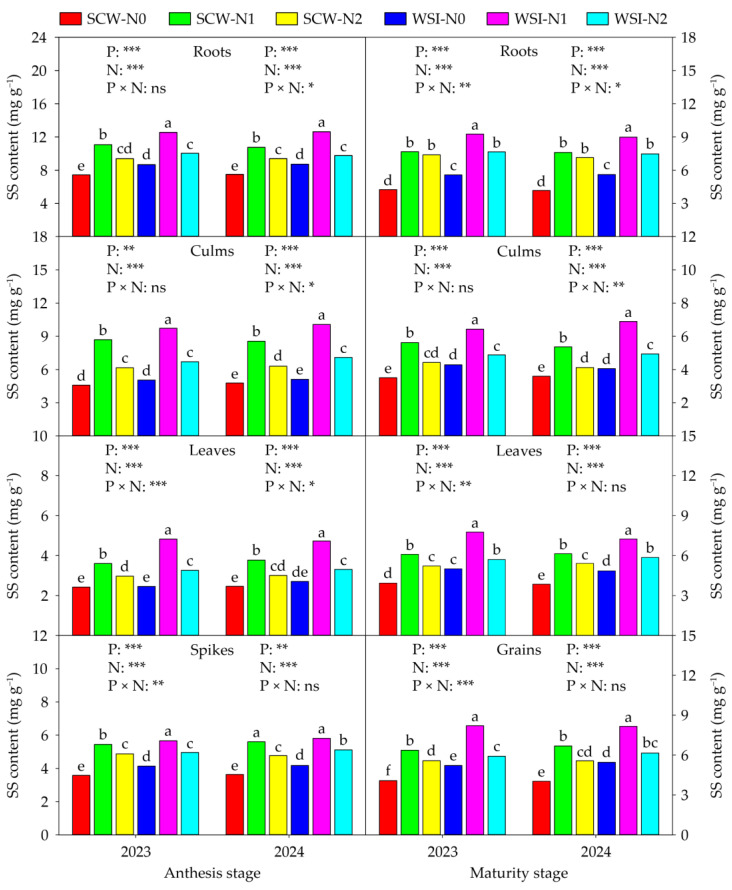
Effects of planting pattern and nitrogen application rate on the soluble sugar (SS) content in waxy sorghum. Data are the mean of three replicates and different lowercase letters within an organ in the same growth stage and year indicate significant differences among treatments at the 0.05 level. SCW: Sole cropped waxy sorghum; WSI: Waxy sorghum intercropped with soybean; N0: Zero nitrogen; N1: Medium nitrogen; N2: High nitrogen; P: Planting pattern; N: Nitrogen application rate; P × N: Interaction between planting pattern and nitrogen application rate. ns, *, **, and *** indicate not significant and significant at the 0.05, 0.01, and 0.001 levels, respectively.

**Figure 4:**
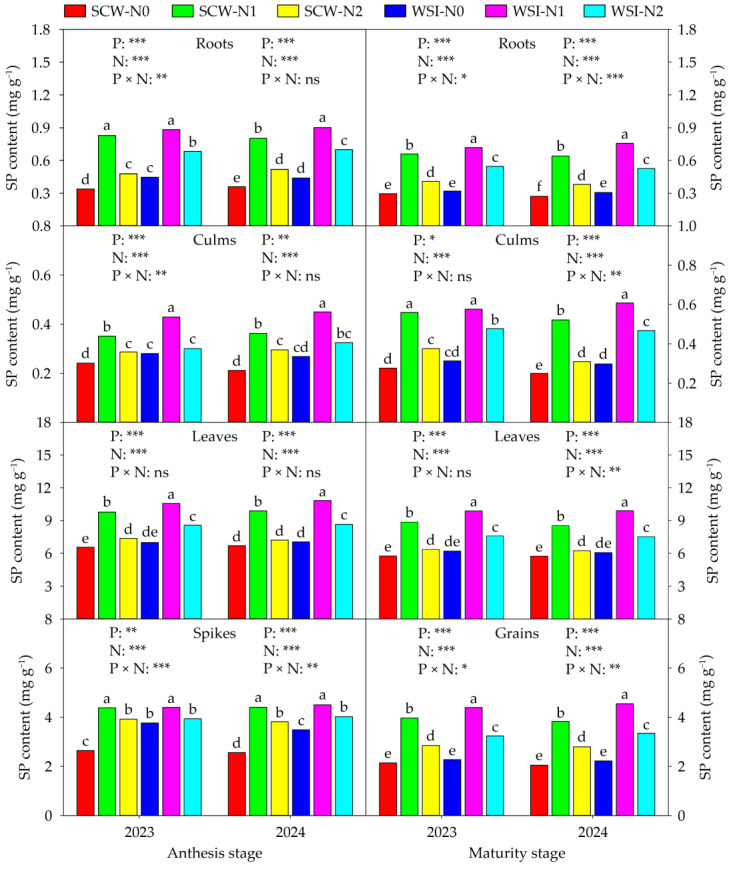
Effects of planting pattern and nitrogen application rate on the soluble protein (SP) content in waxy sorghum. Data are the mean of three replicates and different lowercase letters within an organ in the same growth stage and year indicate significant differences among treatments at the 0.05 level. SCW: Sole cropped waxy sorghum; WSI: Waxy sorghum intercropped with soybean; N0: Zero nitrogen; N1: Medium nitrogen; N2: High nitrogen; P: Planting pattern; N: Nitrogen application rate; P × N: Interaction between planting pattern and nitrogen application rate. ns, *, **, and *** indicate not significant and significant at the 0.05, 0.01, and 0.001 levels, respectively.

**Figure 5:**
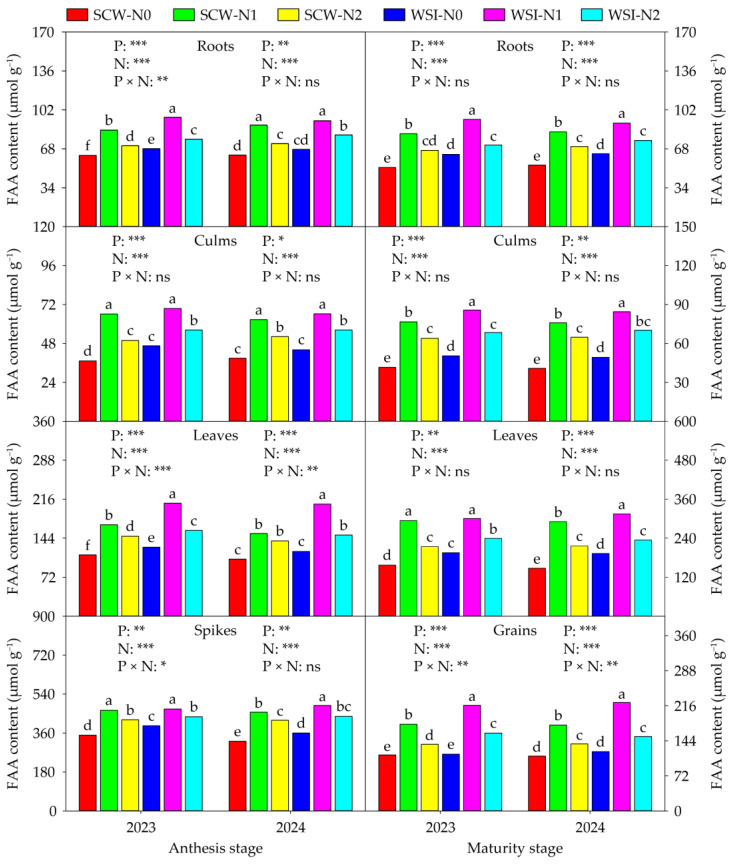
Effects of planting pattern and nitrogen application rate on the free amino acid (FAA) content in waxy sorghum. Data are the mean of three replicates and different lowercase letters within an organ in the same growth stage and year indicate significant differences among treatments at the 0.05 level. SCW: Sole cropped waxy sorghum; WSI: Waxy sorghum intercropped with soybean; N0: Zero nitrogen; N1: Medium nitrogen; N2: High nitrogen; P: Planting pattern; N: Nitrogen application rate; P × N: Interaction between planting pattern and nitrogen application rate. ns, *, **, and *** indicate not significant and significant at the 0.05, 0.01, and 0.001 levels, respectively.

The SS (Figure 3), SP (Figure 4), and FAA (Figure 5) contents of each organ were significantly affected by planting pattern and nitrogen application rate in both stages and both years, and also affected by interaction between planting pattern and nitrogen application rate. Across years and treatments, at the anthesis stage, the orders of SS, SP, and FAA contents among organs were roots > culms > spikes > leaves, leaves > spikes > roots > culms, and spikes > leaves > roots > culms, respectively. At the maturity stage, the SS content among organs was in the sequence of roots > grains > leaves > culms, and the SP and FAA contents among organs were in the order of leaves > grains > roots > culms in both years and all treatments. In addition, in both stages and both years, the SS, SP, and FAA contents of each organ among planting patterns and nitrogen application rates were in the sequence of WSI > SCW and N1 > N2 > N0, respectively.

### 3.4. Yield, Yield Components, and LER

The planting pattern and nitrogen application rate had significant effects on the grain yields of waxy sorghum and soybean, grain weight per spike, and 1000-grain weight of waxy sorghum in both years, while the interaction between planting pattern and nitrogen application rate only had significant effect on the grain yield of waxy sorghum in both years (Table 4). For the mean of two years, the grain yield of waxy sorghum under the WSI treatment was 14.00% higher than that under the SCW treatment, while the grain yield of soybean under the WSI treatment was 77.86% lower than that under the SCS treatment. In addition, compared with the N0 treatment, the N1 and N2 treatments increased the grain yield of waxy sorghum (mean of two years) by, respectively, 13.79% and 7.17%, and increased the grain yield of soybean (mean of two years) by, respectively, 19.08% and 6.89%. In both years, the grain weight per spike and 1000-grain weight of waxy sorghum among planting patterns and nitrogen application rates were in the order of WSI > SCW and N1 > N2 > N0, respectively. Additionally, the LER under the WSI treatment with three nitrogen application rates was greater than 1 in both years, and its maximum value appeared in the WSI-N1 treatment, which was 1.41 in 2023 and 1.44 in 2024.

### 3.5. Nitrogen Use Efficiency

The NUE, NAE, NAPE, NRE, NPFP, and NCR were markedly influenced by planting pattern and nitrogen application rate in both years, while the interaction between planting pattern and nitrogen application rate only had significant effects on the NUE and NRE in both years, and NCR in 2023 (Table 5). Compared to the SCW treatment, the WSI treatment increased the mean NUE, NAE, NAPE, NRE, NPFP, and NCR of two years by 13.78%, 113.87%, 29.20%, 22.04%, 5.95%, and 128.91%, respectively. In addition, for the mean of two years, the NUE, NAE, NAPE, NRE, NPFP, and NCR under the N1 treatment were, respectively, 131.72%, 291.22%, 251.89%, 287.03%, 112.43%, and 86.49% higher than those under the N2 treatment.

### 3.6. PLS-PM Analysis

**Figure 6:**
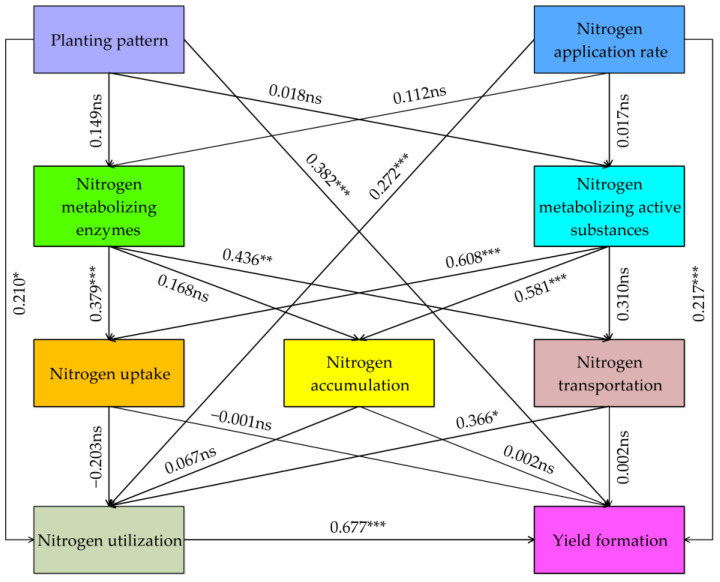
Partial least squares path modeling (PLS-PM) analysis among planting pattern and nitrogen application rate with nitrogen uptake, nitrogen accumulation, nitrogen transportation, nitrogen metabolism physiology, nitrogen utilization, and yield formation in waxy sorghum. The goodness-of-fit (GoF) index is 0.605. Values near the arrows represent the path coefficients between latent variables. ns, *, **, and *** indicate not significant and significant at the 0.05, 0.01, and 0.001 levels, respectively.

As shown in Figure 6, planting pattern and nitrogen application rate had significant and direct positive effects on nitrogen utilization and yield formation. Nitrogen metabolizing enzymes had significant and direct positive effects on nitrogen uptake and nitrogen transportation. Nitrogen metabolizing active substances had significant and direct positive effects on nitrogen uptake and nitrogen accumulation. Nitrogen transportation had a significant and direct positive effect on nitrogen utilization, and nitrogen utilization had a significant and direct positive effect on yield formation.

## 4. Discussion

Nitrogen uptake, accumulation, and transportation are important processes of the nitrogen cycle in plants, which play a key role in improving nitrogen use efficiency and promoting yield formation in crops [15,35]. In cereal–legume intercropping systems, legume crops can promote nitrogen uptake, accumulation, and transportation by cereal crops [36]. Ramirez-Garcia et al. [37] found that barley intercropped with vetch increased the nitrogen concentration and nitrogen accumulation amount of aboveground plants in barley. Nasar et al. [38] discovered that maize intercropped with alfalfa increased the nitrogen content of leaves and grains in maize by, respectively, 42% and 27%, and increased the total nitrogen uptake of maize by 64%. Ahmed et al. [39] indicated that maize intercropped with soybean significantly increased the total nitrogen accumulation of maize under the potassium application rate of 80 kg ha^−1^ for maize and 60 kg ha^−1^ for soybean. Similarly, our study showed that the NC and NA of each organ in waxy sorghum under the WSI treatment were higher than those under the SCW treatment in both stages and both years (Table 2; Figure 2). The following two reasons may explain the increases in NC and NA. On the one hand, the improvement of the plant light environment of waxy sorghum in the waxy sorghum–soybean intercropping system promoted the nitrogen synthesis in various organs of waxy sorghum [29]. On the other hand, the enhancement of the biological nitrogen fixation ability of soybean in the waxy sorghum–soybean intercropping system accelerated the transfer of nitrogen from soybean to waxy sorghum [40]. In this study, the WSI treatment increased the NTA, NTR, and GCRNT of each organ in waxy sorghum compared with the SCW treatment (Table 3), which was consistent with our previous study [30]. These results imply that waxy sorghum intercropped soybean can coordinate the nitrogen flow in source and reservoir of waxy sorghum. Our study showed that the NC, NA, NTA, NTR, and GCRNT of each organ in waxy sorghum among nitrogen application rates were in the order of N1 > N2 > N0 (Table 2 and Table 3; Figure 2), which was similar to a previous finding of Abunyewa et al. [41], who reported that the nitrogen concentration and uptake of stover and grains in sorghum were increased by applying nitrogen fertilizer. This result suggests that nitrogen application can promote nitrogen absorption, accumulation, and transportation in various organs of waxy sorghum, and the medium nitrogen application rate has the best performance. Therefore, waxy sorghum intercropped with soybean combined with medium nitrogen application can increase the nitrogen uptake and accumulation among organs and promote the nitrogen transportation from vegetative organs to grains in waxy sorghum.

Numerous studies have shown that cereal–legume intercropping can enhance the activities of nitrogen metabolizing enzymes in cereal crops. For instance, Nasar et al. [42] discovered that maize intercropped with soybean significantly improved the NR, NiR, and GOGAT activities of leaves in maize as compared with sole cropped maize. Suryapani et al. [43] found that wheat intercropped with lentil increased the NR, NiR, GS, and GOGAT activities of leaves in wheat compared to sole cropped wheat. Liu et al. [44] showed that wheat intercropped with faba bean increased the activities of total GS and GOGAT activities and enhanced the gene expressions of GS1, GS2, Fd-GOGAT, and NADH-GOGAT in the flag leaves of wheat as compared with sole cropped wheat. Likewise, our study observed that the activities of NR, NiR, GS, GOGAT, GDH, and GPT of each organ in waxy sorghum under the WSI treatment were higher than those under the SCW treatment in both stages and both years (Tables S3–S5; Figures S2–S4). In this study, the WSI treatment increased the contents of SS, SP, and FAA of each organ in waxy sorghum in both stages and both years, compared with the SCW treatment (Figure 3, Figure 4 and Figure 5), which was similar to a previous finding of Dang et al. [45], who reported that proso millet intercropped with mung bean increased the SP and FAA contents in lag leaves of proso millet. The increments in the activities of NR, NiR, GS, GOGAT, GDH, and GPT, and contents of SS, SP, and FAA in waxy sorghum under the WSI treatment might be attributed to the following two possibilities. First, the biological nitrogen fixation capacity of soybean enhanced the nitrogen metabolism capacity of waxy sorghum plants by improving the nitrogen content of each organ in waxy sorghum [40]. In addition, it might be due to the improvement of rhizosphere soil physicochemical properties and enzyme activities of waxy sorghum in waxy sorghum–soybean intercropping systems triggering the plant nitrogen metabolism system of waxy sorghum [31]. In the present study, we found that the activities of NR, NiR, GS, GOGAT, GDH, and GPT, and contents of SS, SP, and FAA in each organ of waxy sorghum in both stages and both years increased with the increase in nitrogen application rate from N0 to N1, while decreased with the increase in nitrogen application rate from N1 to N2 (Tables S3–S5; Figures S2–S4 and Figure 3, Figure 4 and Figure 5). Similarly, Nasar et al. [42] showed that the NR, NiR and, GOGAT activities in leaves of maize increased with the increase in nitrogen application rate up to the optimum rate (250 kg ha^−1^), while showing the negative response above the nitrogen application rate of 250 kg ha^−1^. Rehman et al. [46] stated that nitrogen application increased the activities of NR, GS, and GOGAT and the content of SP in leaves of ramie, and 420 kg ha^−1^ of nitrogen fertilizer rates showed the best performance. This result implies that appropriate nitrogen application can enhance the plant nitrogen metabolism capacity of waxy sorghum. Thus, waxy sorghum intercropped with soybean combined with medium nitrogen application can help improve the plant nitrogen uptake, accumulation, and transportation of waxy sorghum by enhancing its nitrogen metabolism capacity.

Intercropping plays an active role in increasing nitrogen use efficiency in crops. Bouras et al. [23] reported that durum wheat intercropped with chickpea significantly increased the nitrogen use efficiency of durum wheat and chickpea under rain-fed mediterranean conditions. Gitari et al. [47] found that potato intercropped with dolichos, pea, and bean improved the nitrogen use efficiency of potato as compared with sole cropped potato. Likewise, in the present study, the NUE, NAE, NAPE, NRE, NPFP, and NCR of waxy sorghum under the WSI treatment were higher than those under the SCW treatment in both years (Table 5). This result might be due to the waxy sorghum–soybean intercropping, which could be helpful to improve the soil microbial community structure and increase the number of nitrogen-fixing bacteria in the soil, thus improving the nitrogen absorption ability of waxy sorghum from the soil, which needs further study. In addition to intercropping, the nitrogen application rate also affected the nitrogen use efficiency of crops. In this study, the NUE, NAE, NAPE, NRE, NPFP, and NCR of waxy sorghum among nitrogen application rates were in the order of N1 > N2 in both years (Table 5), which might be due to the appropriate nitrogen application effectively stimulating the plant nitrogen metabolism system, and then promoting the nitrogen absorption, accumulation and transportation in various organs of waxy sorghum. These results indicate that waxy sorghum intercropped with soybean combined with medium nitrogen application can increase the nitrogen use efficiency of waxy sorghum. Various researchers have reported that cereal–legume intercropping systems, combined with appropriate nitrogen application, improved the production of cereal crops and land-use efficiency [48,49]. Similarly, this study showed that the grain yield, grain weight per spike, and 1000-grain weight of waxy sorghum among planting patterns and nitrogen application rates were, respectively, in the order of WSI > SCW and N1 > N2 > N0, and the LER among treatments was in the sequence of WSI-N1 > WSI-N2 > WSI-N0 in both years (Table 4), which has been confirmed by our previous study [40]. As is well known, the liquor quality is closely related to the grain-related traits of waxy sorghum [50]. In this study, the higher 1000-grain weight and SP content in grains of waxy sorghum under the WSI-N1 treatment can effectively improve the cooking resistance of grains in waxy sorghum and palatability of liquor, respectively. Consequently, waxy sorghum intercropped with soybean, combined with medium nitrogen application, can help enhance the nitrogen uptake and utilization of waxy sorghum by improving nitrogen metabolizing enzymes’ activities and active substances’ contents, thereby promoting its productivity.

This study clarified the nitrogen uptake and utilization characteristics of waxy sorghum under different nitrogen application rates in a waxy sorghum–soybean intercropping system, which is helpful to formulate the application strategy of nitrogen fertilizer in a waxy sorghum–soybean intercropping system. However, our study is limited in that it was conducted for only two years and on one experimental site. We will conduct longer-term and multi-ecological regional studies in the future.

## 5. Conclusions

Our study revealed that waxy sorghum intercropped with soybean combined with medium nitrogen application can help enhance the nitrogen uptake and utilization of waxy sorghum by improving nitrogen metabolizing enzymes’ activities and active substances’ contents, thereby promote their productivity. These findings are helpful to formulate the application strategy of nitrogen fertilizer in waxy sorghum–soybean intercropping systems. Future research will focus on the interaction between waxy sorghum and soybean, such as soil nutrient conversion, soil microbial recruitment, and the allelopathy of roots exudates.

## Figures and Tables

**Table 1:** The fertilization strategies for experimental treatments.

Treatment	Waxy Sorghum			Soybean		
	N (kg ha^−1^)	P_2_O_5_ (kg ha^−1^)	K_2_O (kg ha^−1^)	N (kg ha^−1^)	P_2_O_5_ (kg ha^−1^)	K_2_O (kg ha^−1^)
SCW-N0	0	100	300	/	/	/
SCW-N1	200	100	300	/	/	/
SCW-N2	400	100	300	/	/	/
SCS-N0	/	/	/	0	60	40
SCS-N1	/	/	/	60	60	40
SCS-N2	/	/	/	120	60	40
WSI-N0	0	110	330	0	18	12
WSI-N1	220	110	330	18	18	12
WSI-N2	440	110	330	36	18	12

SCW: Sole cropped waxy sorghum; SCS: Sole cropped soybean; WSI: Waxy sorghum intercropped with soybean; N0: Zero nitrogen; N1: Medium nitrogen; N2: High nitrogen.

**Table 2:** Effects of planting pattern and nitrogen application rate on the nitrogen content (NC, g kg^−1^) in waxy sorghum.

Year	Treatment	Anthesis Stage				Maturity Stage			
		Roots	Culms	Leaves	Spikes	Roots	Culms	Leaves	Grains
2023	SCW-N0	5.39 e	4.06 e	21.37 f	14.45 e	3.99 f	2.56 f	19.80 e	12.89 d
	SCW-N1	7.23 b	6.52 b	26.48 b	16.30 c	5.10 c	3.89 c	24.43 b	14.60 bc
	SCW-N2	6.48 c	5.61 c	23.74 d	16.00 c	4.63 d	3.52 d	22.14 c	14.22 c
	WSI-N0	6.01 d	4.95 d	22.53 e	15.08 d	4.25 e	3.11 e	20.81 d	13.22 d
	WSI-N1	7.94 a	7.36 a	28.12 a	19.11 a	5.47 a	4.33 a	25.22 a	15.60 a
	WSI-N2	7.65 a	6.71 b	25.39 c	16.94 b	5.31 b	4.04 b	22.58 c	15.10 ab
	Source of variation								
	P	***	***	***	***	***	***	***	***
	N	***	***	***	***	***	***	***	***
	P × N	*	ns	ns	***	**	ns	ns	ns
2024	SCW-N0	5.25 e	3.98 f	21.04 f	14.24 f	4.02 e	2.49 f	19.20 f	12.48 f
	SCW-N1	7.24 b	6.23 c	25.99 b	16.52 c	5.04 b	3.74 c	23.61 b	14.48 c
	SCW-N2	6.42 c	5.57 d	23.55 d	15.74 d	4.60 c	3.42 d	21.45 d	13.94 d
	WSI-N0	6.07 d	4.84 e	22.19 e	15.18 e	4.35 d	2.99 e	20.24 e	13.42 e
	WSI-N1	7.93 a	7.14 a	28.25 a	19.27 a	5.53 a	4.17 a	25.27 a	15.70 a
	WSI-N2	7.28 b	6.54 b	25.25 c	16.79 b	5.13 b	3.95 b	22.52 c	14.92 b
	Source of variation								
	P	***	***	***	***	***	***	***	***
	N	***	***	***	***	***	***	***	***
	P × N	ns	ns	*	***	ns	ns	**	ns

Data are the mean of three replicates and different lowercase letters within an organ in the same growth stage and year indicate significant differences among treatments at the 0.05 level. SCW: Sole cropped waxy sorghum; WSI: Waxy sorghum intercropped with soybean; N0: Zero nitrogen; N1: Medium nitrogen; N2: High nitrogen; P: Planting pattern; N: Nitrogen application rate; P × N: Interaction between planting pattern and nitrogen application rate. ns, *, **, and *** indicate not significant and significant at the 0.05, 0.01, and 0.001 levels, respectively.

**Table 3:** Effects of planting pattern and nitrogen application rate on the nitrogen transportation in waxy sorghum.

Year	Treatment	NTA (kg ha^−1^)			NTR (%)			GCRNT (%)		
		Roots	Culms	Leaves	Roots	Culms	Leaves	Roots	Culms	Leaves
2023	SCW-N0	4.06 e	12.77 e	15.21 e	36.74 e	48.72 b	20.15 c	5.09 e	16.01 d	19.10 c
	SCW-N1	7.22 c	25.51 c	25.77 bc	42.59 bc	52.85 a	23.19 abc	7.02 bc	24.84 b	25.13 b
	SCW-N2	5.73 d	18.97 d	20.27 de	40.29 d	48.99 b	21.40 c	6.34 cd	20.99 c	22.42 bc
	WSI-N0	5.70 d	17.80 d	21.06 cd	40.59 cd	49.70 b	22.07 bc	6.09 d	19.00 c	22.51 bc
	WSI-N1	10.41 a	35.16 a	38.16 a	44.72 a	53.82 a	26.50 a	8.29 a	27.98 a	30.36 a
	WSI-N2	8.57 b	28.25 b	29.79 b	43.71 ab	52.49 a	25.83 ab	7.41 ab	24.39 b	25.72 ab
	Source of variation									
	P	***	***	***	***	*	**	***	***	*
	N	***	***	***	***	**	*	***	***	**
	P × N	*	*	ns	ns	ns	ns	ns	ns	ns
2024	SCW-N0	3.49 d	11.91 e	14.65 d	32.94 d	47.65 d	19.95 d	4.47 d	15.26 f	18.73 d
	SCW-N1	7.06 b	23.18 c	26.25 b	42.18 ab	51.46 bc	23.88 abc	6.96 ab	22.82 c	25.87 b
	SCW-N2	5.54 c	18.30 d	20.51 c	40.04 bc	48.82 d	22.86 bcd	6.24 bc	20.61 d	23.09 bc
	WSI-N0	5.58 c	17.68 d	20.36 c	39.35 c	50.07 cd	22.07 cd	5.83 c	18.50 e	21.30 cd
	WSI-N1	9.62 a	34.45 a	39.19 a	42.71 a	54.46 a	26.52 a	7.72 a	27.65 a	31.44 a
	WSI-N2	7.63 b	28.20 b	28.97 b	41.74 abc	52.74 ab	25.36 ab	6.69 b	24.74 b	25.41 b
	Source of variation									
	P	***	***	***	**	***	*	**	***	**
	N	***	***	***	***	**	**	***	***	***
	P × N	ns	***	*	**	ns	ns	ns	ns	ns

Data are the mean of three replicates and different lowercase letters within an organ in the same growth stage and year indicate significant differences among treatments at the 0.05 level. SCW: Sole cropped waxy sorghum; WSI: Waxy sorghum intercropped with soybean; N0: Zero nitrogen; N1: Medium nitrogen; N2: High nitrogen; P: Planting pattern; N: Nitrogen application rate; P × N: Interaction between planting pattern and nitrogen application rate; NTA: Nitrogen transportation amount before anthesis; NTR: Nitrogen transportation rate before anthesis; GCRNT: Contribution rate of nitrogen transportation before anthesis to grains. ns, *, **, and *** indicate not significant and significant at the 0.05, 0.01, and 0.001 levels, respectively.

**Table 4:** Effects of planting pattern and nitrogen application rate on the grain yields of waxy sorghum and soybean, yield components of waxy sorghum, and land equivalent ratio (LER).

Year	Treatment	Grain Yield (kg ha^−1^)		Yield Components of Waxy Sorghum		LER
		Waxy Sorghum	Soybean	Grain Weight per Spike (g)	1000-Grain Weight (g)	
2023	SCW-N0	4783.15 f	/	55.11 d	17.87 d	/
	SCW-N1	5282.64 c	/	61.04 b	22.37 ab	/
	SCW-N2	4889.87 e	/	58.67 bc	21.33 b	/
	SCS-N0	/	2153.88 c	/	/	/
	SCS-N1	/	2365.72 a	/	/	/
	SCS-N2	/	2236.85 b	/	/	/
	WSI-N0	5168.47 d	397.06 f	57.79 cd	19.06 c	1.26 b
	WSI-N1	6020.66 a	635.74 d	65.22 a	23.14 a	1.41 a
	WSI-N2	5750.85 b	477.49 e	61.83 b	21.98 b	1.39 a
	Source of variation					
	P	***	***	**	**	/
	N	***	***	***	***	/
	P × N	***	ns	ns	ns	/
2024	SCW-N0	4798.97 d	/	54.39 d	17.75 e	/
	SCW-N1	5217.08 c	/	60.95 b	22.50 ab	/
	SCW-N2	4922.65 d	/	58.58 c	21.10 cd	/
	SCS-N0	/	2173.09 c	/	/	/
	SCS-N1	/	2487.91 a	/	/	/
	SCS-N2	/	2305.15 b	/	/	/
	WSI-N0	5181.41 c	417.73 e	56.90 c	19.85 d	1.27 c
	WSI-N1	6159.81 a	633.52 d	64.51 a	23.18 a	1.44 a
	WSI-N2	5797.41 b	476.32 e	61.49 b	21.84 bc	1.38 b
	Source of variation					
	P	***	***	***	**	/
	N	***	***	***	***	/
	P × N	***	ns	ns	ns	/

Data are the mean of three replicates and different lowercase letters within a column in the same year indicate significant differences among treatments at the 0.05 level. SCW: Sole cropped waxy sorghum; SCS: Sole cropped soybean; WSI: Waxy sorghum intercropped with soybean; N0: Zero nitrogen; N1: Medium nitrogen; N2: High nitrogen; P: Planting pattern; N: Nitrogen application rate; P × N: Interaction between planting pattern and nitrogen application rate. ns, **, and *** indicate not significant and significant at the 0.01 and 0.001 levels, respectively.

**Table 5:** Effects of planting pattern and nitrogen application rate on the nitrogen use efficiency in waxy sorghum.

Year	Treatment	NUE (kg kg^−1^)	NAE (kg kg^−1^)	NAPE (%)	NRE (%)	NPFP (kg kg^−1^)	NCR (%)
2023	SCW-N1	1.05 b	2.50 b	11.55 b	28.71 b	26.41 b	9.45 b
	SCW-N2	0.46 d	0.27 d	2.64 d	7.73 c	12.22 d	2.18 c
	WSI-N1	1.19 a	3.87 a	14.55 a	34.39 a	27.37 a	14.15 a
	WSI-N2	0.52 c	1.32 c	5.04 c	9.26 c	13.07 c	10.13 b
	Source of variation						
	P	***	***	**	**	***	***
	N	***	***	***	***	***	***
	P × N	**	ns	ns	*	ns	**
2024	SCW-N1	1.04 b	2.09 b	11.75 a	28.52 b	26.09 b	8.01 b
	SCW-N2	0.44 d	0.31 c	2.66 c	6.78 d	12.31 c	2.51 c
	WSI-N1	1.19 a	4.45 a	13.19 a	34.96 a	28.00 a	15.83 a
	WSI-N2	0.51 c	1.40 b	4.17 b	8.93 c	13.18 c	10.62 b
	Source of variation						
	P	***	***	*	***	**	***
	N	***	***	***	***	***	***
	P × N	***	ns	ns	**	ns	ns

Data are the mean of three replicates and different lowercase letters within a column in the same year indicate significant differences among treatments at the 0.05 level. SCW: Sole cropped waxy sorghum; WSI: Waxy sorghum intercropped with soybean; N1: Medium nitrogen; N2: High nitrogen; P: Planting pattern; N: Nitrogen application rate; P × N: Interaction between planting pattern and nitrogen application rate; NUE: Nitrogen uptake efficiency; NAE: Nitrogen agronomy efficiency; NAPE: Nitrogen apparent efficiency; NRE: Nitrogen recovery efficiency; NPFP: Nitrogen partial factor productivity; NCR: Nitrogen contribution rate. ns, *, **, and *** indicate not significant and significant at the 0.05, 0.01, and 0.001 levels, respectively.

## Data Availability

The original contributions presented in this study are included in the article and Supplementary Materials. Further inquiries can be directed to the corresponding author.

## Supplementary Materials

The following supporting information can be downloaded at: https://www.mdpi.com/article/10.3390/plants14091384/s1, Table S1: Analysis of variance (ANOVA) for the effects of planting pattern and nitrogen application rate on the dry mater accumulation amount (DMA) in waxy sorghum; Table S2: Analysis of variance (ANOVA) for the effects of planting pattern and nitrogen application rate on the nitrogen accumulation amount (NA) in waxy sorghum; Table S3: Effects of planting pattern and nitrogen application rate on the nitrate reductase (NR) activity (U g^−1^) in waxy sorghum; Table S4: Effects of planting pattern and nitrogen application rate on the nitrite reductase (NiR) activity (U g^−1^) in waxy sorghum; Table S5: Effects of planting pattern and nitrogen application rate on the glutamine synthetase (GS) activity (U g^−1^) in waxy sorghum; Figure S1: The daily mean air temperature and precipitation during the two growing seasons in 2023 and 2024; Figure S2: Effects of planting pattern and nitrogen application rate on the glutamate synthetase (GOGAT) activity in waxy sorghum; Figure S3: Effects of planting pattern and nitrogen application rate on the glutamate dehydrogenase (GDH) activity in waxy sorghum; Figure S4: Effects of planting pattern and nitrogen application rate on the glutamic-pyruvic transaminase (GPT) activity in waxy sorghum.
