# Supplementary material for: Effects of Nitrogen Application Rate on Nitrogen Uptake and Utilization in Waxy Sorghum Under Waxy Sorghum–Soybean Intercropping Systems
Source: Plants (Basel). 2025 May 3;14(9):1384. doi: 10.3390/plants14091384 (PMC12073204; doi:10.3390/plants14091384)
Supplement: Supplementary file 1 [file plants-14-01384-s001.zip › plants-3586402-supplementary.pdf]

Table S1. Analysis of variance (ANOVA) for the effects of planting pattern and nitrogen application rate on the dry mater accumulation amount (DMA) in waxy sorghum.

Year	Source of variation	Anthesis stage				Maturity stage			
		Roots	Culms	Leaves	Spikes	Roots	Culms	Leaves	Grains
2023	P	***	***	***	***	***	***	***	***
	N	***	***	***	***	***	***	***	***
	P × N	**	***	*	ns	**	***	ns	ns
2024	P	***	***	***	***	***	***	***	***
	N	***	***	***	***	***	***	***	***
	P × N	***	***	***	***	***	***	***	**

P: Planting pattern; N: Nitrogen application rate; P × N: Interaction between planting pattern and nitrogen application rate. ns, *, **, and *** indicate not significant and significant at the 0.05, 0.01, and 0.001 levels, respectively.

Table S2. Analysis of variance (ANOVA) for the effects of planting pattern and nitrogen application rate on the nitrogen accumulation amount (NA) in waxy sorghum.

Year	Source of variation	Anthesis stage				Maturity stage			
		Roots	Culms	Leaves	Spikes	Roots	Culms	Leaves	Grains
2023	P	***	***	***	***	***	***	***	***
	N	***	***	***	***	***	***	***	***
	P × N	**	**	**	*	**	**	*	**
2024	P	***	***	***	***	***	***	***	***
	N	***	***	***	***	***	***	***	***
	P × N	**	***	***	***	***	**	***	**

P: Planting pattern; N: Nitrogen application rate; P × N: Interaction between planting pattern and nitrogen application rate. ns, *, **, and *** indicate not significant and significant at the 0.05, 0.01, and 0.001 levels, respectively.

Table S3. Effects of planting pattern and nitrogen application rate on the nitrate reductase (NR) activity (U g^{-1}) in waxy sorghum.

Year	Treatment	Anthesis stage				Maturity stage			
		Roots	Culms	Leaves	Spikes	Roots	Culms	Leaves	Grains
2023	SCW-N0	4.54 d	0.36 d	14.56 e	5.75 e	3.82 e	0.98 d	15.18 e	1.29 d
	SCW-N1	7.72 a	1.16 a	21.06 b	9.01 a	6.43 b	1.53 ab	22.09 b	2.25 a
	SCW-N2	6.42 bc	0.56 c	17.06 d	7.53 c	4.84 d	1.34 c	18.14 d	1.88 b
	WSI-N0	5.59 c	0.44 d	15.69 de	6.48 d	4.55 d	1.10 d	18.86 cd	1.55 c
	WSI-N1	8.42 a	1.23 a	22.64 a	9.35 a	7.69 a	1.64 a	25.64 a	2.32 a
	WSI-N2	6.67 b	0.70 b	18.91 c	8.31 b	5.40 c	1.45 bc	20.40 bc	1.98 b
	Source of variation								
	P	*	**	**	**	***	**	***	*
	N	***	***	***	***	***	***	***	***
	P × N	ns	ns	ns	ns	ns	ns	ns	ns
2024	SCW-N0	4.61 e	0.38 e	14.49 e	5.96 e	3.91 e	0.96 e	15.13 d	1.24 d
	SCW-N1	7.61 b	1.21 b	21.80 b	8.97 ab	6.45 b	1.64 b	22.03 b	2.36 a
	SCW-N2	6.21 c	0.55 d	16.64 d	7.29 cd	4.76 d	1.40 c	17.61 c	1.90 bc
	WSI-N0	5.51 d	0.47 d	15.90 de	6.55 de	4.69 d	1.21 d	17.94 c	1.63 c
	WSI-N1	8.52 a	1.38 a	23.78 a	9.32 a	7.64 a	1.83 a	25.92 a	2.41 a
	WSI-N2	6.78 c	0.76 c	19.23 c	8.23 bc	5.35 c	1.57 b	19.55 c	2.06 b
	Source of variation								
	P	***	***	***	*	***	***	***	*
	N	***	***	***	***	***	***	***	***
	P × N	ns	ns	ns	ns	ns	ns	ns	ns

Data are the mean of three replicates and different lowercase letters within an organ in the same growth stage and year indicate significant differences among treatments at the 0.05 level. SCW: Sole cropped waxy sorghum; WSI: Waxy sorghum intercropped with soybean; N0: Zero nitrogen; N1: Medium nitrogen; N2: High nitrogen; P: Planting pattern; N: Nitrogen application rate; P × N: Interaction between planting pattern and nitrogen application rate. ns, *, **, and *** indicate not significant and significant at the 0.05, 0.01, and 0.001 levels, respectively.

Table S4. Effects of planting pattern and nitrogen application rate on the nitrite reductase (NiR) activity (U g^{-1}) in waxy sorghum.

Year	Treatment	Anthesis stage				Maturity stage			
		**Roots**	**Culms**	**Leaves**	**Spikes**	**Roots**	**Culms**	**Leaves**	**Grains**
2023	SCW-N0	0.32 e	0.28 e	0.80 e	0.66 e	0.18 f	0.14 e	0.93 e	0.54 e
	SCW-N1	0.73 b	0.46 b	1.60 b	0.93 b	0.47 b	0.44 b	1.69 b	0.98 b
	SCW-N2	0.49 d	0.37 cd	1.16 c	0.70 de	0.28 d	0.24 d	1.18 cd	0.76 c
	WSI-N0	0.35 e	0.33 d	0.94 d	0.76 cd	0.23 e	0.17 e	1.04 de	0.63 d
	WSI-N1	0.99 a	0.55 a	1.89 a	1.04 a	0.54 a	0.50 a	2.02 a	1.13 a
	WSI-N2	0.58 c	0.41 bc	1.22 c	0.82 c	0.36 c	0.31 c	1.33 c	0.82 c
	Source of variation								
	P	***	***	***	**	***	**	***	***
	N	***	***	***	***	***	***	***	***
	P × N	**	ns	*	ns	ns	ns	ns	ns
2024	SCW-N0	0.35 e	0.29 e	0.82 f	0.65 e	0.17 e	0.16 e	0.90 e	0.52 e
	SCW-N1	0.74 b	0.48 b	1.63 b	0.94 b	0.51 b	0.42 b	1.72 b	0.97 b
	SCW-N2	0.52 d	0.40 cd	1.14 d	0.69 de	0.30 d	0.22 d	1.16 d	0.75 cd
	WSI-N0	0.40 e	0.35 de	0.98 e	0.74 d	0.24 de	0.19 de	1.07 d	0.65 d
	WSI-N1	1.01 a	0.58 a	1.84 a	1.07 a	0.59 a	0.51 a	2.05 a	1.15 a
	WSI-N2	0.59 c	0.47 bc	1.23 c	0.83 c	0.38 c	0.34 c	1.35 c	0.85 c
	Source of variation								
	P	***	**	***	***	**	***	***	**
	N	***	***	***	***	***	***	***	***
	P × N	***	ns	ns	ns	ns	*	ns	ns

Data are the mean of three replicates and different lowercase letters within an organ in the same growth stage and year indicate significant differences among treatments at the 0.05 level. SCW: Sole cropped waxy sorghum; WSI: Waxy sorghum intercropped with soybean; N0: Zero nitrogen; N1: Medium nitrogen; N2: High nitrogen; P: Planting pattern; N: Nitrogen application rate; P × N: Interaction between planting pattern and nitrogen application rate. ns, *, **, and *** indicate not significant and significant at the 0.05, 0.01, and 0.001 levels, respectively.

Table S5. Effects of planting pattern and nitrogen application rate on the glutamine synthetase (GS) activity (U g^{-1}) in waxy sorghum.

Year	Treatment	Anthesis stage				Maturity stage			
		Roots	Culms	Leaves	Spikes	Roots	Culms	Leaves	Grains
2023	SCW-N0	7.25 e	2.02 e	62.97 e	20.73 e	4.67 f	2.83 f	71.49 e	18.60 d
	SCW-N1	10.40 b	4.84 b	101.49 b	34.78 b	8.54 b	6.44 b	113.84 b	26.69 b
	SCW-N2	8.87 d	2.77 d	80.72 d	26.66 d	6.33 d	4.27 d	93.98 d	21.60 c
	WSI-N0	8.55 d	2.59 d	79.13 d	22.82 e	5.67 e	3.75 e	88.05 d	22.48 c
	WSI-N1	11.29 a	5.57 a	118.40 a	40.25 a	9.92 a	8.38 a	122.85 a	31.66 a
	WSI-N2	9.50 c	3.51 c	91.71 c	29.45 c	7.40 c	5.32 c	104.44 c	25.74 b
	Source of variation								
	P	***	***	***	***	***	***	***	***
	N	***	***	***	***	***	***	***	***
	P × N	ns	ns	ns	ns	ns	*	ns	ns
2024	SCW-N0	7.48 e	2.04 f	64.49 d	21.12 d	4.80 f	2.87 f	73.35 e	18.50 d
	SCW-N1	10.21 b	4.88 b	99.91 b	33.26 b	8.43 b	6.51 b	108.93 ab	27.00 b
	SCW-N2	8.85 cd	2.82 d	81.25 c	27.93 c	6.43 d	4.37 d	92.20 cd	21.53 c
	WSI-N0	8.59 d	2.47 e	75.91 c	23.30 d	5.69 e	3.68 e	87.94 d	22.16 c
	WSI-N1	11.65 a	5.70 a	112.07 a	40.49 a	10.40 a	8.43 a	116.92 a	33.11 a
	WSI-N2	9.45 bc	3.67 c	93.84 b	30.41 bc	7.62 c	5.29 c	100.86 bc	26.38 b
	Source of variation								
	P	***	***	***	**	***	***	**	***
	N	***	***	***	***	***	***	***	***
	P × N	ns	**	ns	ns	ns	*	ns	ns

Data are the mean of three replicates and different lowercase letters within an organ in the same growth stage and year indicate significant differences among treatments at the 0.05 level. SCW: Sole cropped waxy sorghum; WSI: Waxy sorghum intercropped with soybean; N0: Zero nitrogen; N1: Medium nitrogen; N2: High nitrogen; P: Planting pattern; N: Nitrogen application rate; P × N: Interaction between planting pattern and nitrogen application rate. ns, *, **, and *** indicate not significant and significant at the 0.05, 0.01, and 0.001 levels, respectively.

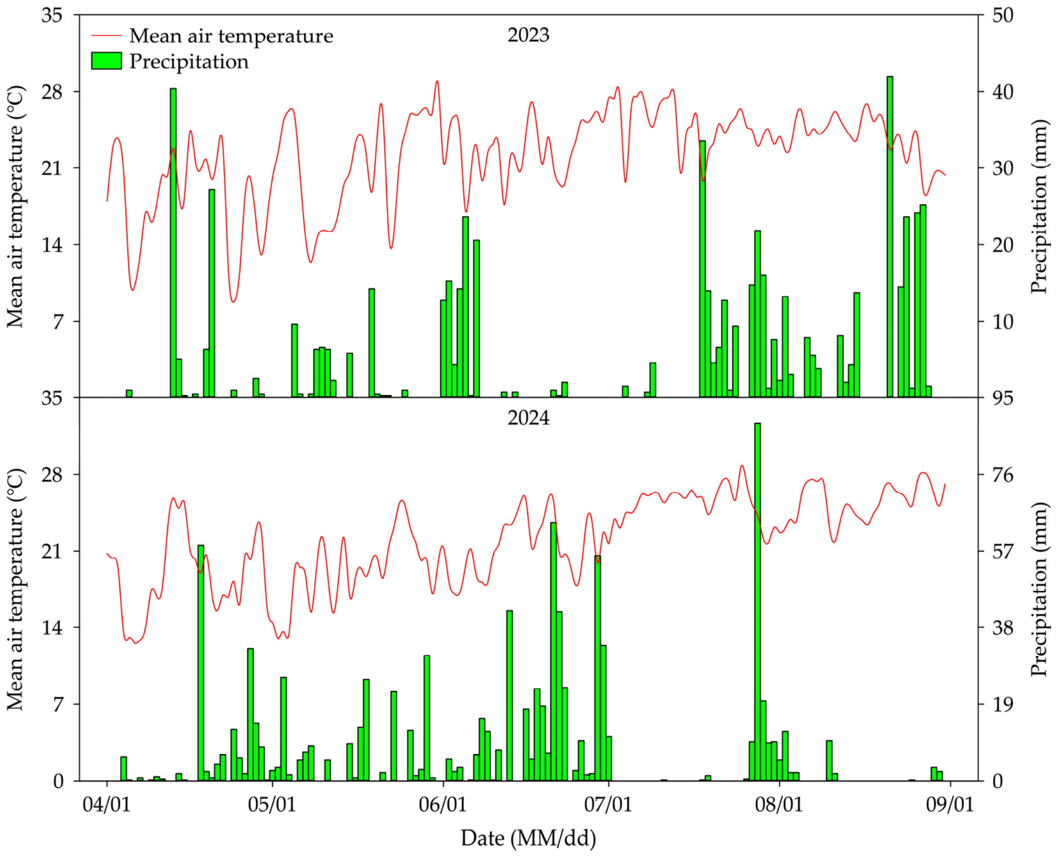

Figure S1. The daily mean air temperature and precipitation during the two growing seasons in 2023 and 2024.

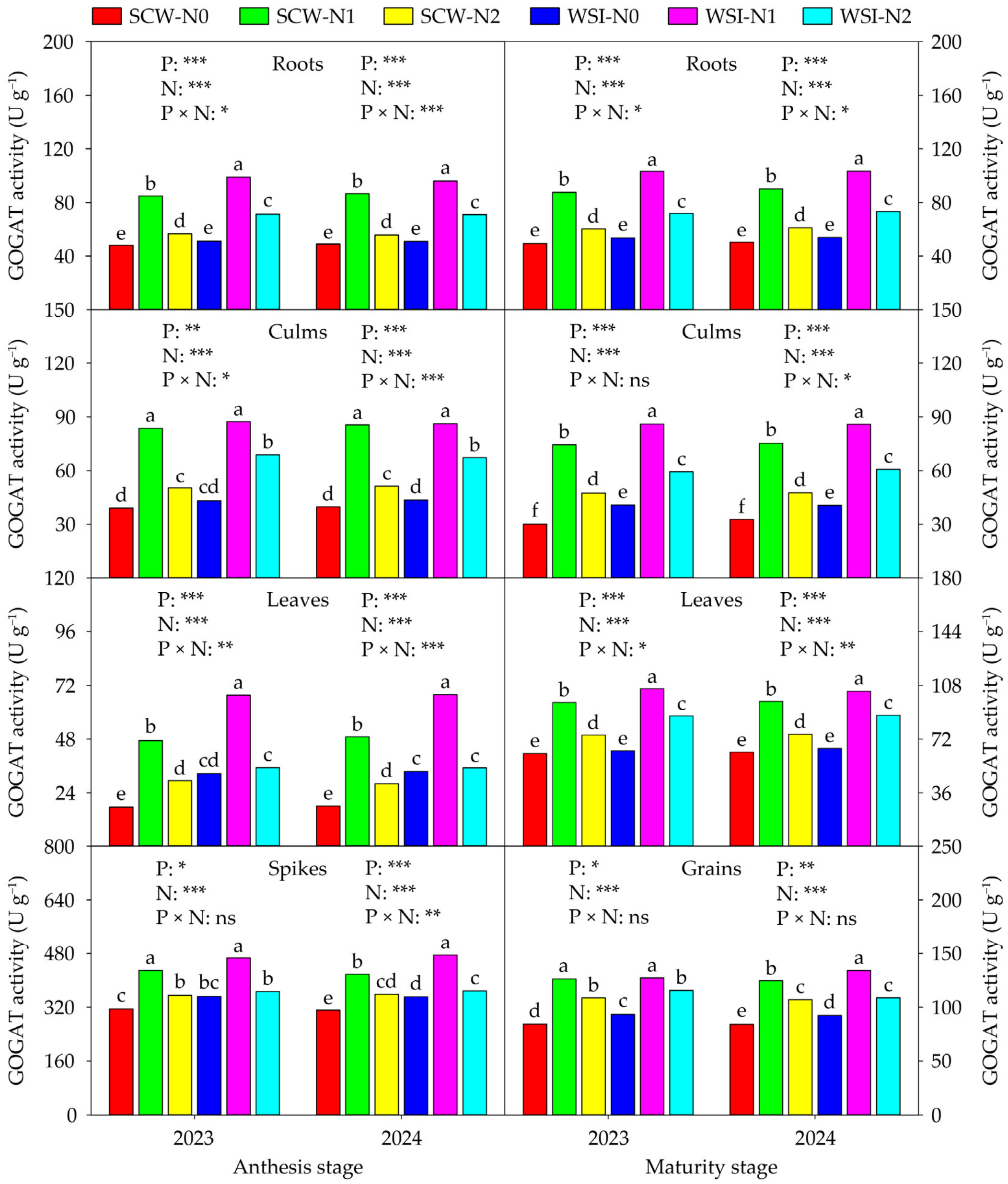

Figure S2. Effects of planting pattern and nitrogen application rate on the glutamate synthetase (GOGAT) activity in waxy sorghum. Data are the mean of three replicates and different lowercase letters within an organ in the same growth stage and year indicate significant differences among treatments at the 0.05 level. SCW: Sole cropped waxy sorghum; WSI: Waxy sorghum intercropped with soybean; N0: Zero nitrogen; N1: Medium nitrogen; N2: High nitrogen; P: Planting pattern; N: Nitrogen application rate; P × N: Interaction between planting pattern and nitrogen application rate. ns, *, **, and *** indicate not significant and significant at the 0.05, 0.01, and 0.001 levels, respectively.

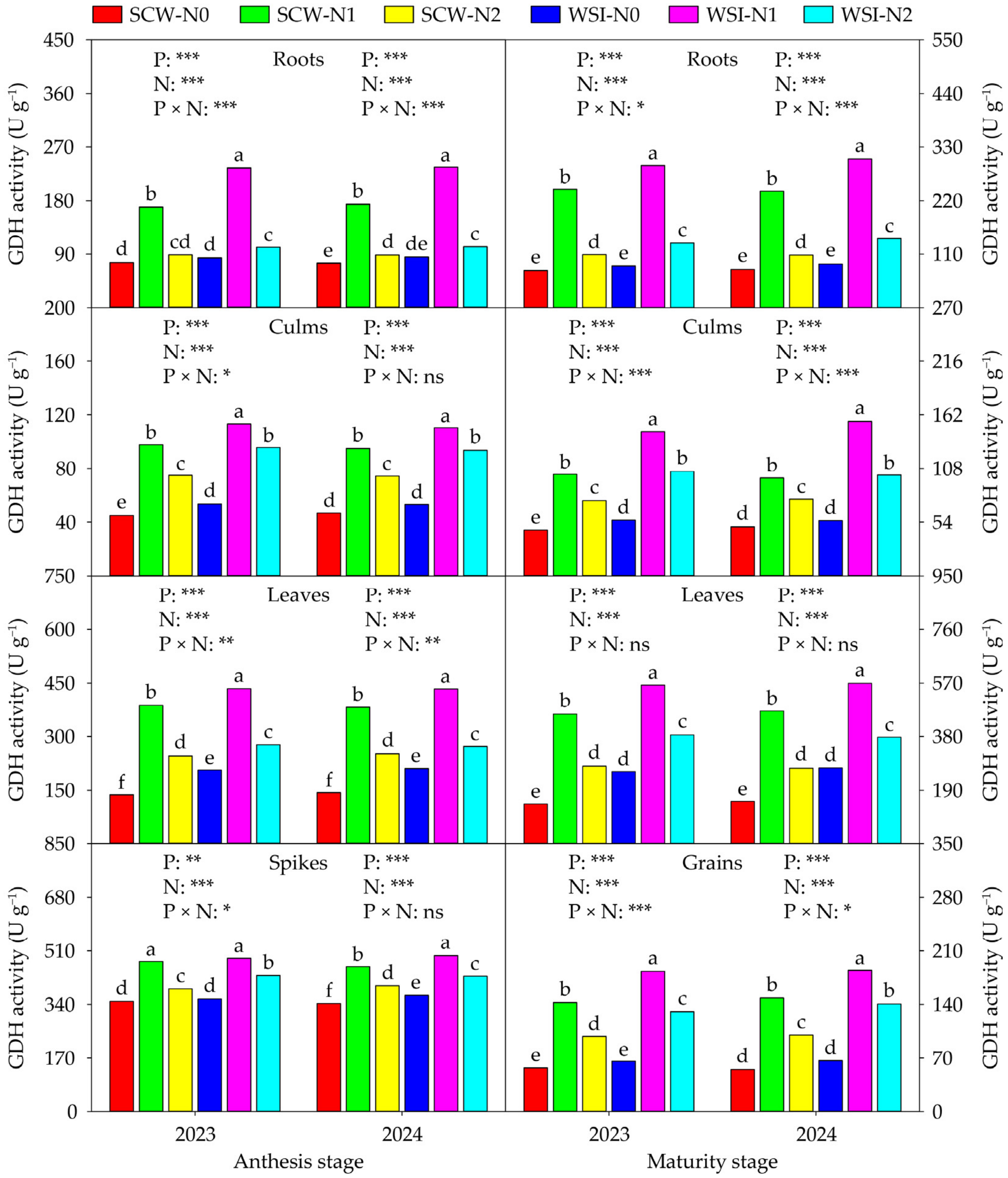

Figure S3. Effects of planting pattern and nitrogen application rate on the glutamate dehydrogenase (GDH) activity in waxy sorghum. Data are the mean of three replicates and different lowercase letters within an organ in the same growth stage and year indicate significant differences among treatments at the 0.05 level. SCW: Sole cropped waxy sorghum; WSI: Waxy sorghum intercropped with soybean; N0: Zero nitrogen; N1: Medium nitrogen; N2: High nitrogen; P: Planting pattern; N: Nitrogen application rate; P × N: Interaction between planting pattern and nitrogen application rate. ns, *, **, and *** indicate not significant and significant at the 0.05, 0.01, and 0.001 levels, respectively.

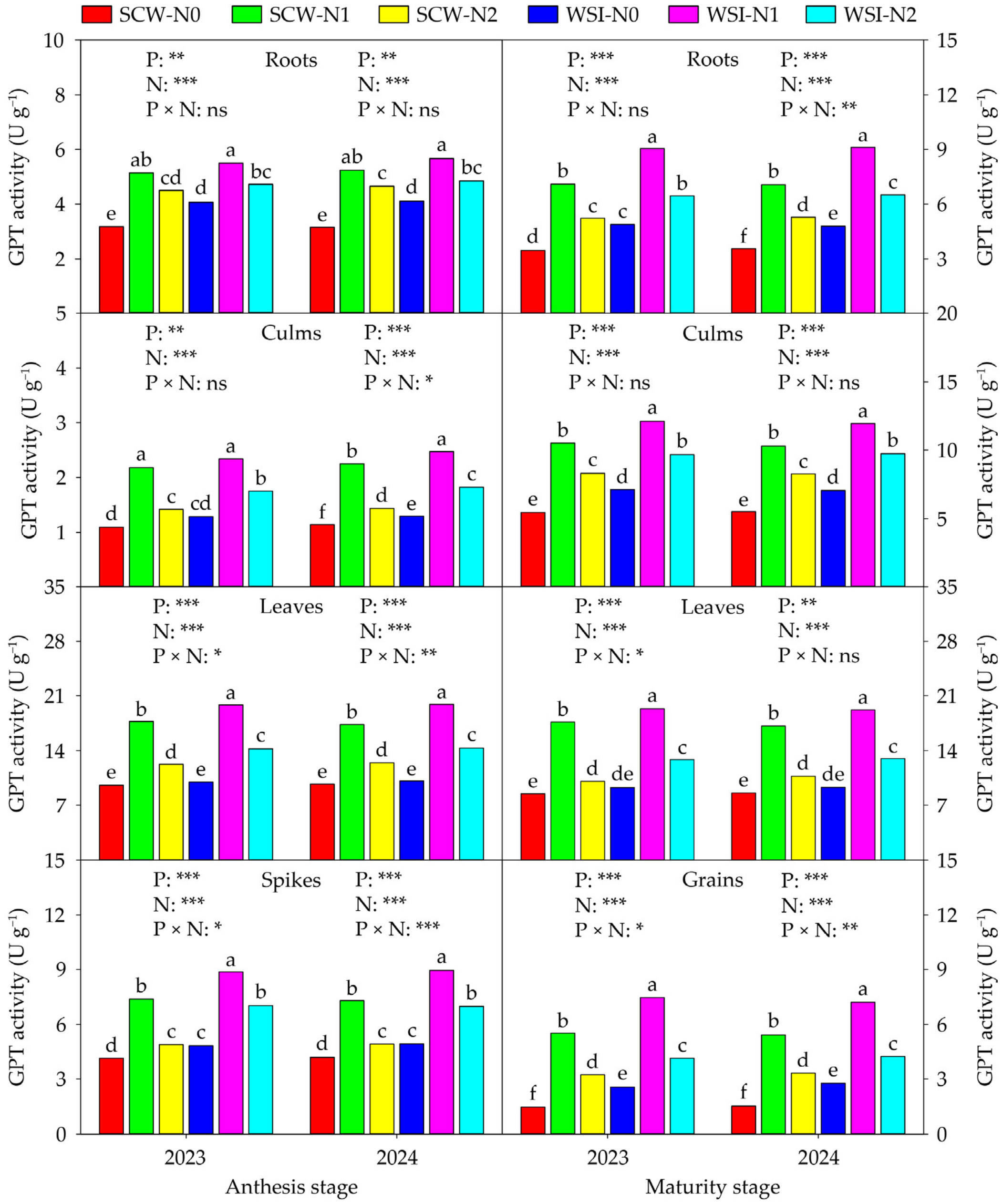

Figure S4. Effects of planting pattern and nitrogen application rate on the glutamic-pyruvic transaminase (GPT) activity in waxy sorghum. Data are the mean of three replicates and different lowercase letters within an organ in the same growth stage and year indicate significant differences among treatments at the 0.05 level. SCW: Sole cropped waxy sorghum; WSI: Waxy sorghum intercropped with soybean; N0: Zero nitrogen; N1: Medium nitrogen; N2: High nitrogen; P: Planting pattern; N: Nitrogen application rate; P × N: interaction between planting pattern and nitrogen application rate. ns, *, **, and *** indicate not significant and significant at the 0.05, 0.01, and 0.001 levels, respectively.
